# Explainable AI for chest radiographs: sex-stratified fairness auditing in CNN-based pneumonia detection

**DOI:** 10.1038/s41598-026-62761-3

**Published:** 2026-07-20

**Authors:** Matteo Haupt, Elena Jolkver, Anne Schwerk

**Affiliations:** 1 Department of Diagnostic and Interventional Radiology, Carl Von Ossietzky Universität Oldenburg, Oldenburg, Germany; 2 IU Internationale Hochschule GmbH, Erfurt, Germany

**Keywords:** Artificial intelligence, Deep learning, Algorithmic fairness, Explainability (XAI), Chest radiography, Pneumonia, Computational biology and bioinformatics, Diseases, Health care, Mathematics and computing, Medical research

## Abstract

Artificial intelligence (AI) systems for chest X-ray (CXR) interpretation can achieve high standalone diagnostic accuracy, yet limited transparency raises concerns about subgroup disparities. We audited sex-stratified performance and attention patterns in CNN-based pneumonia detection using the RSNA Pneumonia Detection Challenge dataset (26,684 frontal adult CXRs). ImageNet-pretrained InceptionV3 models were trained across five prespecified random seeds and combined as a seed-ensemble. Analyses used a prespecified, fixed, patient-level sex- and label-balanced test set (n = 1,000; 250 pneumonia-positive and 250 pneumonia-negative per sex) and a single validation-derived operating point held constant across sex. Baseline performance was robust (AUROC 0.859 [0.836, 0.881], Brier 0.155 [0.141, 0.169]). Males showed higher discrimination than females (AUROC 0.882 vs 0.837, gap + 0.045 [+ 0.002, + 0.091]) and a lower false-positive rate (FPR gap − 0.073 [− 0.145, − 0.006]). To contextualize disparities, we quantified Grad-CAM attention relative to lung segmentation masks; in-lung activation was lower in females (60.8% vs 64.7%, M − F + 3.9 pp [+ 1.6, + 6.4]). A combined mitigation (group-balanced sampling + adversarial debiasing) preserved performance (AUROC 0.864 [0.843, 0.885]) while reducing AUROC/specificity gaps and attenuating the false-positive disparity. Quantitative explainability can complement fairness auditing and mitigation in medical imaging.

**Supplementary Information:**

The online version contains supplementary material available at 10.1038/s41598-026-62761-3.

## Introduction

Artificial intelligence (AI), particularly deep learning with convolutional neural networks (CNNs), has become a key enabler of automated image interpretation in medical imaging. Within this domain, chest X-ray (CXR) remains one of the most frequently performed and widely accessible diagnostic examinations worldwide^1,2^. Accurate CXR interpretation is essential for detecting a broad range of thoracic conditions, including pneumonia, tuberculosis, and lung cancer^3^. However, CXRs are inherently challenging to interpret: disease manifestations such as pneumonia may be subtle or atypical, and substantial inter-reader variability persists, particularly in high-volume or resource-constrained clinical settings^4^. These characteristics not only motivate the use of automated decision support systems but also increase the risk that diagnostic errors, whether human or algorithmic, are unevenly distributed across patient subgroups if models are trained and evaluated without subgroup-aware analysis.

Recent years have seen pretrained CNN architectures achieve expert-level performance for diagnostic tasks on large, annotated CXR datasets^5^. However, despite strong benchmark performance, the widespread clinical adoption of AI systems remains limited, with a prevailing concern being the lack of transparency in how these “black-box” models arrive at their decisions. Explainable artificial intelligence (XAI) techniques have therefore become essential not only for clinical trust and workflow integration, but also for systematic validation of model behavior, error analysis, and the identification of subgroup-dependent biases^6,7^. Importantly, explainability methods also enable the systematic auditing of model behavior across demographic subgroups, thereby linking interpretability directly to fairness assessment. For example, Yang et al. showed that CNNs can recover recorded patient sex from CXR images with high accuracy^8^. Such sex-correlated features are a fairness concern because a model may use them as shortcuts rather than the pulmonary findings of pneumonia, which can skew error rates between male and female patients.

Numerous XAI methods have been introduced in CXR analysis, ranging from accessible saliency maps to more advanced approaches such as Gradient-weighted Class Activation Mapping (Grad-CAM). Saliency maps compute the gradient of the model’s output with respect to input pixels, yielding rapid and computationally inexpensive heatmaps; however, their spatial imprecision often limits clinical usefulness^9^. Grad-CAM offers improved visualizations by linking activations with class-specific gradients, resulting in localization within meaningful anatomical regions and broad compatibility with modern CNNs^10,11^. However, visual plausibility alone does not guarantee that model attention aligns with clinically relevant or fair decision-making. Yet the systematic evaluation of XAI techniques remains challenging. Existing studies employ a variety of evaluation strategies, including region-overlap metrics, pointing-game analyses, qualitative expert review, and fidelity-based perturbation methods^11,12^. Each approach offers complementary insights but is associated with trade-offs in reproducibility, clinical validity, and resource requirements. This methodological heterogeneity highlights the need for reproducible, quantitative XAI metrics that can be systematically compared across subgroups. Notably, such evaluation strategies are rarely applied in a subgroup-stratified manner, limiting their ability to explain why performance disparities arise.

A critical but sometimes underappreciated challenge in medical AI is algorithmic fairness, i.e. the absence of algorithmic bias^13^. In clinical AI, fairness is commonly operationalized as comparable performance, error rates, and calibration across clinically relevant subgroups. Algorithmic bias refers to systematic errors that disproportionately affect groups defined by characteristics such as recorded sex, age, or ethnicity. It can arise from dataset imbalances, acquisition artifacts, or model design and may be amplified by training and evaluation practices^14^. For example, imbalanced datasets or subgroup-agnostic evaluation can conceal systematic error patterns that only emerge at deployment. In the context of CXR-based pneumonia detection, several studies have demonstrated that sex bias is both common and consequential: models frequently underperform for women, especially when trained on imbalanced or non-representative datasets^15–17^. Such disparities are clinically consequential and underscore the need for evaluation frameworks that explicitly assess both performance and error distributions across sex^18^. If such differences are not explicitly accounted for, AI systems risk reinforcing existing diagnostic inequities rather than alleviating them.

While prior work has examined subgroup performance disparities and, separately, explainability methods in chest radiography, few studies have integrated quantitative explainability with subgroup-stratified performance evaluation to probe the mechanisms underlying observed disparities. In particular, existing analyses rarely connect attention localization patterns to specific error types (e.g., false positives or false negatives) under controlled, subgroup-balanced evaluation settings. This gap limits both mechanistic understanding and the design of targeted mitigation strategies.

This study addresses these gaps by empirically investigating sex-specific disparities in CNN-based pneumonia detection on CXRs. Adopting an integrated fairness–explainability framework, we specifically assess: (i) how XAI methods reveal and contextualize sex-specific performance disparities; (ii) the data- and model-level mechanisms associated with these disparities, such as attention localization; and (iii) the efficacy of fairness-aware mitigation strategies in reducing performance gaps without degrading overall utility.

## Results

**Fig. 1:**
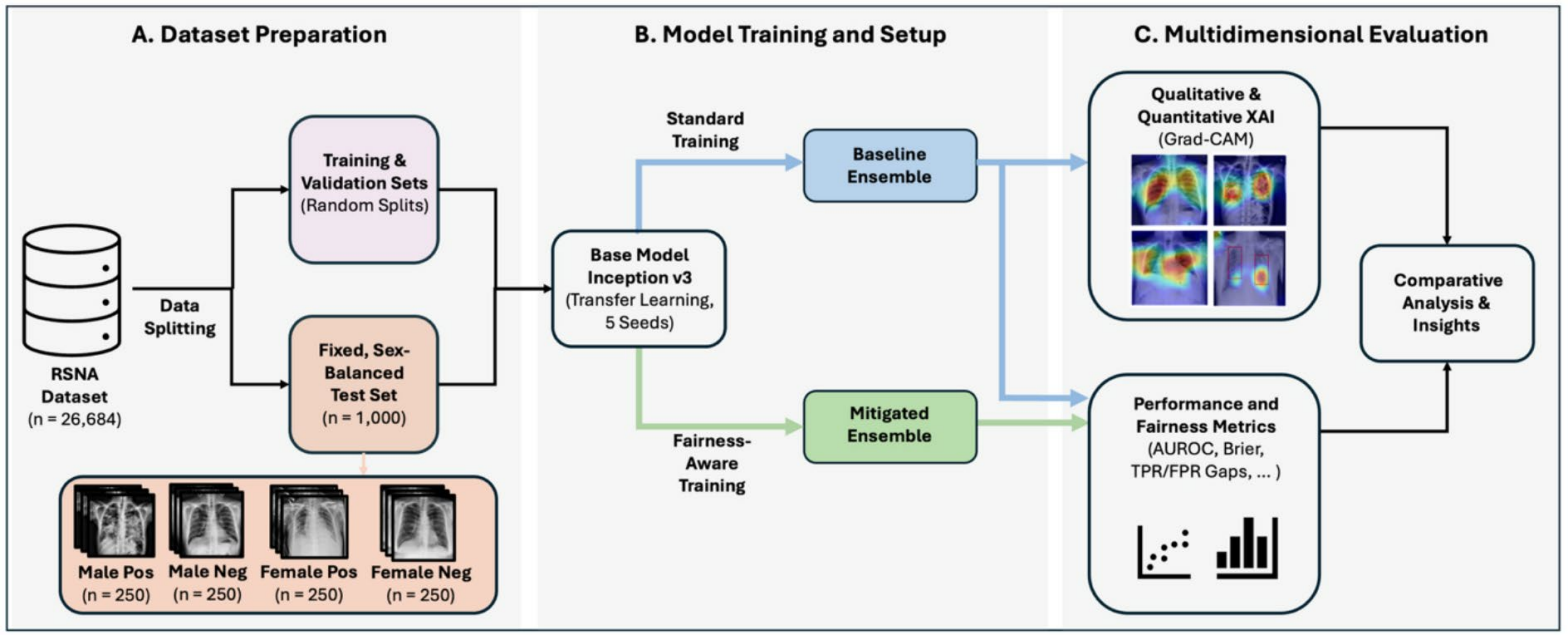
Overview of the study design and analysis pipeline. (**A**) Dataset preparation: RSNA dataset (n = 26,684) with creation of a fixed, sex-balanced test set (n = 1,000). (**B**) Model training: InceptionV3 trained across five seeds to form baseline and fairness-mitigated ensembles. (**C**) Multidimensional evaluation: Performance, fairness metrics, and Grad-CAM–based explainability with lung segmentation.

All analyses were performed on the prespecified, fixed sex- and label-balanced test set (n = 1,000; 250 pneumonia-positive and 250 pneumonia-negative radiographs per sex). Model training was repeated across five independent random seeds, and all reported estimates correspond to the seed-ensemble, obtained by averaging predicted probabilities across seeds at the patient level (Fig. 1).

## Global performance of the baseline and mitigation ensembles

The baseline InceptionV3 seed-ensemble demonstrated strong overall discrimination and good probabilistic performance on the balanced test set (Table 1). The AUROC was 0.859 (95% CI 0.836–0.881) and the area under the precision–recall curve (PR-AUC) was 0.861 (95% CI 0.830–0.886). At the fixed operating point (t = 0.462), balanced accuracy was 0.777 (95% CI 0.751–0.803), with sensitivity of 0.810 (95% CI 0.776–0.843) and specificity of 0.744 (95% CI 0.707–0.784). Calibration, assessed using the Brier score, was 0.155 (95% CI 0.141–0.169). Reliability analysis showed mild miscalibration before post-hoc scaling, which improved after temperature scaling (Figure S3). The combined fairness-aware mitigation strategy preserved global performance. At the fixed operating point (t = 0.425) the mitigation ensemble achieved an AUROC of 0.864 (95% CI 0.843–0.885) and a PR-AUC of 0.866 (95% CI 0.839–0.891), with balanced accuracy of 0.784 (95% CI 0.760–0.809), sensitivity of 0.832 (95% CI 0.800–0.863), specificity of 0.736 (95% CI 0.697–0.775), and a Brier score of 0.152 (95% CI 0.138–0.166) (Table 1). Ablation analysis (Table S2) revealed that while group-balanced sampling alone reduced the sex-specific AUROC gap to + 0.027 [95% CI − 0.018 to + 0.073], adversarial debiasing independently achieved near-parity with a gap of + 0.003 [95% CI − 0.044 to + 0.049]. The combined approach achieved the highest overall AUROC (0.864) while still reducing the sex AUROC gap (M − F) compared with the baseline model, indicating a performance–fairness trade-off across mitigation components.

**Table 1:** Overall performance of the baseline and mitigation ensembles on the fixed balanced test set. Overall discrimination and classification performance of the baseline and mitigation CNN seed-ensembles on the fixed balanced test set. Ensembles were constructed by averaging predicted probabilities across five prespecified random seeds at the patient level. Point estimates are reported with 95% confidence intervals (CIs) obtained via patient-level, stratified bootstrap resampling. Δ overall denotes the paired difference (mitigation − baseline) with bootstrap 95% CI.

Metric	Baseline ensemble (95% CI)	Mitigation ensemble (95% CI)	Δ overall (Mit–Base) (95% CI)
AUROC	0.859 [0.836, 0.881]	0.864 [0.843, 0.885]	+ 0.005 [− 0.005, + 0.015]
PR-AUC	0.861 [0.830, 0.886]	0.866 [0.839, 0.891]	+ 0.005 [− 0.006, + 0.017]
Balanced accuracy	0.777 [0.751, 0.803]	0.784 [0.760, 0.809]	+ 0.007 [− 0.011, + 0.026]
Brier score	0.155 [0.141, 0.169]	0.152 [0.138, 0.166]	− 0.003 [− 0.009, + 0.004]
Sensitivity	0.810 [0.776, 0.843]	0.832 [0.800, 0.863]	+ 0.022 [− 0.004, + 0.047]
Specificity	0.744 [0.707, 0.784]	0.736 [0.697, 0.775]	− 0.008 [− 0.033, + 0.018]

Calibration was similar under mitigation, with mild miscalibration before scaling that improved after temperature scaling (Figure S5). Paired differences between mitigation and baseline were small for all metrics, and all corresponding 95% confidence intervals included zero, indicating no clear evidence of global performance loss or gain. ROC and precision–recall curves are shown in Figures S1 and S2.

### Sex-stratified performance and sex gaps

Sex-stratified evaluation of the baseline ensemble showed modest performance differences between male and female patients (Table 2). Probabilistic error was lower in males (Brier score 0.140, 95% CI 0.123–0.158) than females (0.170, 95% CI 0.149–0.193), corresponding to a Brier gap of − 0.030 (95% CI − 0.060 to − 0.003). Sex-stratified reliability showed more pronounced miscalibration in females than males before scaling, with heterogeneous subgroup effects after global temperature scaling (Figure S4). In contrast, sensitivity was comparable between sexes (gap + 0.020, 95% CI − 0.049 to + 0.090) (Table 2). After mitigation, results revealed a clear trade-off between different fairness criteria. While disparities in specificity and false positives were effectively reduced, the sensitivity gap widened. Specifically, the AUROC gap decreased from + 0.045 to + 0.026 (95% CI − 0.018 to + 0.069), and the specificity gap decreased from + 0.073 to + 0.025 (95% CI − 0.054 to + 0.099), reflecting a reduction in the disproportionate false-positive rate among females. However, this improvement shifted the disparity toward sensitivity: while sensitivity increased in both sexes, the gain was disproportionately larger in males, resulting in an increased post-mitigation sensitivity (TPR) gap of + 0.056 (95% CI − 0.006 to + 0.122) compared to + 0.020 at baseline. Under mitigation, sex-stratified calibration remained better in males than females before scaling, with heterogeneous subgroup effects after global temperature scaling (Figure S6). Changes in sex gaps are summarized as Δ gap (mitigation minus baseline) in Table 2.

**Table 2:** Sex-stratified performance and sex gap: baseline vs mitigation. Sex-stratified performance of baseline and mitigation CNN ensembles and corresponding sex gaps on the fixed balanced test set. Metrics are reported separately for male and female subgroups with 95% bootstrap CIs derived using patient-level, stratified resampling. The sex gap is defined as M − F (male minus female); positive values indicate higher performance in males (for Brier score, negative values indicate lower error in males). Δ gap reports the change in the sex gap after mitigation (mitigation − baseline).

Metric	Baseline Male (95% CI)	Baseline Female (95% CI)	Baseline gap M − F (95% CI)	Mitigation Male (95% CI)	Mitigation Female (95% CI)	Mitigation gap M − F (95% CI)	Δ gap (Mit − Base)
AUROC	0.882 [0.856, 0.906]	0.837 [0.804, 0.868]	+ 0.045 [+ 0.002, + 0.091]	0.878 [0.847, 0.906]	0.851 [0.818, 0.882]	+ 0.026 [− 0.018, + 0.069]	− 0.019
PR-AUC	0.876 [0.843, 0.907]	0.845 [0.805, 0.882]	+ 0.031 [− 0.020, + 0.088]	0.876 [0.836, 0.910]	0.856 [0.818, 0.892]	+ 0.020 [− 0.033, + 0.070]	− 0.011
Balanced accuracy	0.800 [0.764, 0.835]	0.754 [0.719, 0.790]	+ 0.046 [− 0.001, + 0.098]	0.804 [0.769, 0.837]	0.764 [0.726, 0.800]	+ 0.040 [− 0.009, + 0.091]	− 0.006
Brier score	0.140 [0.123, 0.158]	0.170 [0.149, 0.193]	− 0.030 [− 0.060, − 0.003]	0.142 [0.123, 0.161]	0.163 [0.142, 0.184]	− 0.021 [− 0.050, + 0.007]	+ 0.009
Sensitivity	0.820 [0.778, 0.861]	0.800 [0.751, 0.845]	+ 0.020 [− 0.049, + 0.090]	0.860 [0.816, 0.901]	0.804 [0.755, 0.850]	+ 0.056 [− 0.006, + 0.122]	+ 0.036
Specificity	0.780 [0.726, 0.829]	0.707 [0.650, 0.764]	+ 0.073 [+ 0.006, + 0.145]	0.748 [0.692, 0.803]	0.723 [0.667, 0.778]	+ 0.025 [− 0.054, + 0.099]	− 0.048

**Fig. 2:**
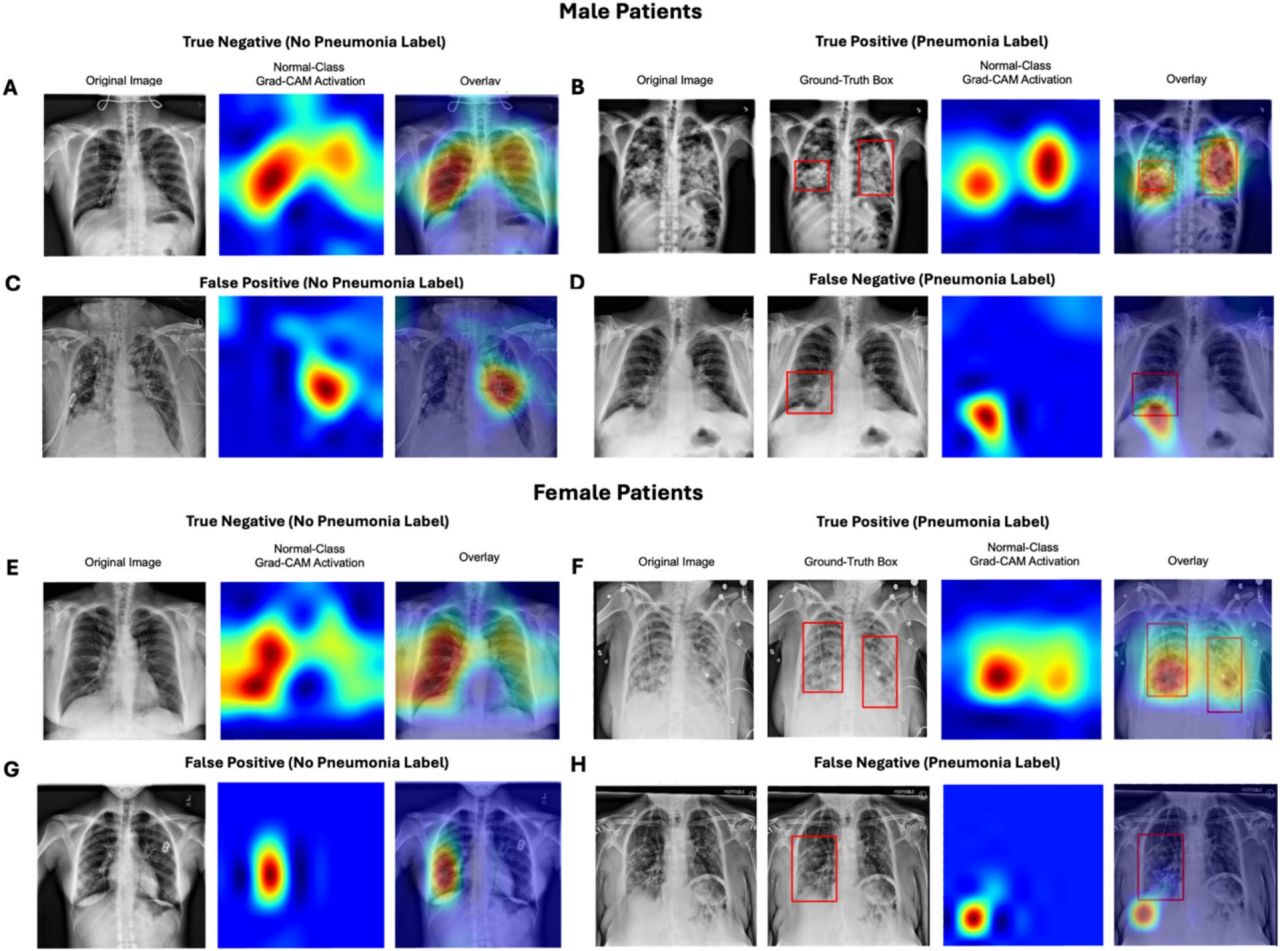
Representative Grad-CAM visualizations by sex and prediction outcome. Representative chest radiographs from male patients (**A**–**D**) and female patients (**E**–**H**) are shown for all four prediction outcomes. Each panel shows the original radiograph, the Grad-CAM heatmap computed for the model-predicted class, and the corresponding overlay. For pneumonia-labelled cases (TP and FN), the expert ground-truth bounding boxes are displayed in red. Color intensity indicates the relative contribution of image regions to the model’s decision for the predicted class (warmer colors = higher contribution). Bounding boxes are shown for reference only and were not used for localization evaluation. Model confidence scores (probability assigned to the predicted class) are: (**A**) 0.98 (TN, predicted Normal), (**B**) 0.64 (TP, predicted Pneumonia), (**C**) 0.76 (FP, predicted Pneumonia), (**D**) 0.64 (FN, predicted Normal), (**E**) 0.97 (TN, predicted Normal), (**F**) 0.86 (TP, predicted Pneumonia), (**G**) 0.57 (FP, predicted Pneumonia), (**H**) 0.55 (FN, predicted Normal).

Representative Grad-CAM visualizations, selected to illustrate high-confidence predictions for true negatives and typical decision-boundary cases for positive and error outcomes, are shown in Fig. 2.

### Fairness gaps

At the fixed operating point, we further quantified classification fairness using rate-based measures (Table 2). True-positive rate (TPR) corresponds to sensitivity, false-negative rate (FNR) to 1 − sensitivity, and false-positive rate (FPR) to 1 − specificity. Sex gaps were defined as male minus female (M − F), with 95% bootstrap confidence intervals.

Under the baseline ensemble, the true-positive rate (TPR) gap (M − F) was + 0.020 (95% CI − 0.049 to + 0.090). In absolute counts (n = 250 pneumonia cases per sex), this corresponded to 205 correctly identified cases in males versus 200 in females. In contrast, the false-positive rate (FPR) gap (M − F) was − 0.073 (95% CI − 0.145 to − 0.006). To translate this into clinical impact (n = 250 non-pneumonia cases per sex), the disparity resulted in 73 false positives in female patients compared to 55 in male patients, indicating a substantially higher burden of false alarms for women. Female false positives also showed the lowest in-lung activation fractions of all subgroups (Table 3), consistent with greater extra-pulmonary attention in these cases.

**Table 3:** Sex-stratified Grad-CAM attention inside anatomical lung fields. Grad-CAM activation was quantified as the proportion of activation mass within lung segmentation masks (“in-lung activation fraction”). Results are reported for the full test set and stratified by prediction outcome (TP/TN/FP/FN) at the fixed operating points. Values show subgroup means; Δ denotes male minus female in percentage points (pp) with 95% confidence intervals from patient-level stratified bootstrap. The analysis uses the seed-ensemble Grad-CAM aggregation to reduce random-initialization variability.

Subset	Male mean (%)	Female mean (%)	Δ (M − F), pp (95% CI)
Overall	64.7	60.8	+ 3.9 [+ 1.6, + 6.4]
True positives	61.8	58.3	+ 3.5 [-1.2, + 8.2]
True negatives	71.5	68.1	+ 3.3 [+ 0.7, + 6.1]
False positives	48.9	42.0	+ 6.9 [-1.6, + 15.3]
False negatives	62.8	60.6	+ 2.2 [-2.7, + 7.1]

Following mitigation, the TPR gap increased to + 0.056 (95% CI − 0.006 to + 0.122), reflecting a widening disparity in detection (215 detected male cases versus 201 female cases). Conversely, the FPR gap was reduced in magnitude to − 0.025 (95% CI − 0.099 to + 0.054). In absolute terms, the number of false positives converged (69 in females vs. 63 in males), effectively mitigating the excess false-positive burden observed at baseline. Overall, mitigation primarily reduced disparities in false-positive rates, while shifts in sensitivity-related gaps highlighted trade-offs between different fairness criteria.

## Quantitative explainability: Grad-CAM attention inside lung fields

**Fig. 3:**
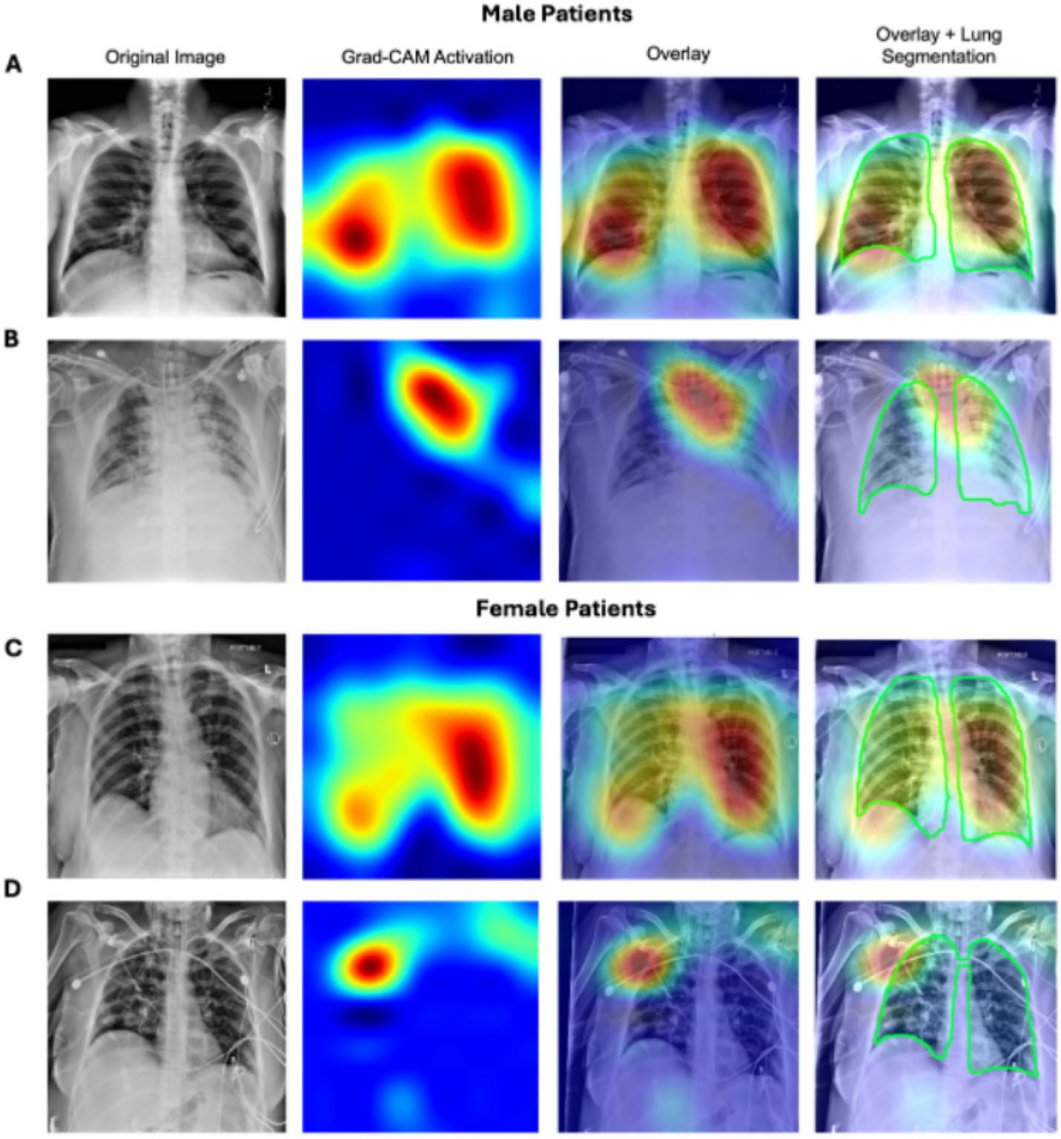
Model attention in relation to lung segmentation masks. Representative chest radiographs from male patients (**A**–**B**) and female patients (**C**–**D**) are shown to illustrate Grad-CAM attention relative to anatomical lung boundaries. Each row displays the original radiograph, the Grad-CAM heatmap computed for the model-predicted class, the corresponding heatmap overlay, and the overlay with the lung segmentation contour (green). Grad-CAM activation was quantified as the fraction of total (normalized) activation mass located inside versus outside the lung segmentation mask, highlighting cases with predominantly pulmonary versus extra-pulmonary attention. Model confidence scores (probability assigned to the predicted class) and in-/out-of-lung activation fractions are: (**A**) TN, predicted Normal: 0.99; 78% inside lungs / 22% outside; (**B**) TP, predicted Pneumonia: 0.69; 38% inside / 62% outside; (**C**) TP, predicted Pneumonia: 0.84; 76% inside / 24% outside; (**D**) TP, predicted Pneumonia: 0.67; 18% inside / 82% outside.

To relate subgroup performance differences to model behavior, Grad-CAM attention was quantified relative to anatomical lung boundaries using segmentation masks (Fig. 3). Across the full test set cohort, the baseline ensemble allocated a higher proportion of activation within the lungs for male patients (64.7%) than for female patients (60.8%), corresponding to a male–female difference (M − F) of + 3.9 percentage points (95% CI + 1.6 to + 6.4) (Table 3).

Directionally consistent differences were observed across prediction outcomes. The difference was most clearly supported for true negatives (+ 3.3 percentage points, 95% CI + 0.7 to + 6.1), while true positives, false positives, and false negatives showed larger uncertainty intervals (Table 3). Notably, false-positive predictions in female patients exhibited the lowest in-lung activation fractions, suggesting an association with increased extra-pulmonary attention at the selected operating point. Representative examples illustrating predominantly pulmonary versus extra-pulmonary attention patterns are shown in Fig. 3.

## Discussion

This study audited sex-stratified fairness in CNN-based pneumonia detection on chest radiographs and complemented subgroup performance evaluation with anatomically grounded, quantitative explainability. Using a prespecified, patient-level sex- and label-balanced test cohort and a single validation-derived operating point held constant across sex, we observed modest but consistent differences favoring male patients, most notably higher discrimination and specificity alongside lower probabilistic error. Although absolute gaps were moderate, reporting them is clinically relevant because global AUROC can mask subgroup-specific burdens and error trade-offs.

To contextualize overall performance, our baseline discrimination aligned with contemporary CNN-based chest radiograph classification pipelines^19,20^. Crucially, our objective was not to maximize leaderboard metrics, but to establish a robust, representative baseline for fairness auditing. By using a standard architecture without excessive hyperparameter tuning, we aimed to reduce the likelihood that the observed sex-specific disparities were driven primarily by architecture-specific optimization choices. In addition, generalization across institutions and acquisition contexts remains a major challenge, and pneumonia models may implicitly learn hospital- or device-specific correlates that inflate internal test performance^21^.

From a clinical perspective, the observed false-positive disparity is not benign: excess false positives in female patients can translate into avoidable downstream imaging, antibiotic exposure, and unnecessary admissions. Prior work has shown that demographic imbalance and subgroup-dependent errors are common in medical imaging AI and can remain hidden under aggregate evaluation^15,16^.

These considerations motivate routine sex-disaggregated reporting of rate-based errors at clinically meaningful thresholds, alongside discrimination and calibration. Quantitative Grad-CAM analysis showed lower in-lung activation fractions in female radiographs than in male radiographs, with the lowest in-lung fractions among female false positives. Importantly, the absolute in-lung percentage should not be interpreted as the share of the decision made inside the lungs. Grad-CAM is explicitly a coarse localization technique and, in practice, saliency maps often exhibit spill-over to borders or high-contrast structures due to limited spatial resolution, upsampling, and gradient-based artifacts—effects that can produce apparent activation along ribs, diaphragmatic contours, mediastinum, and image edges even when the underlying discriminative signal is pulmonary^22^. More broadly, quantitative benchmarks have demonstrated that commonly used saliency methods, including Grad-CAM, frequently show limited localization fidelity in chest X-ray interpretation despite visually plausible heatmaps^23^, and sanity-check frameworks have highlighted failure modes where saliency can be misleading if interpreted naively^24^. Thus, substantial activation outside the lungs may partly reflect known technical limitations of Grad-CAM. The observed sex difference in in-lung activation should therefore be interpreted as an associative and hypothesis-generating auditing signal rather than causal evidence of shortcut learning. In this setting, the comparative finding suggests that subgroup-correlated non-pulmonary image features, such as soft-tissue attenuation, breast shadows, body habitus, or image-border effects, may have contributed to the observed performance differences, particularly among female patients. These candidate explanations were not tested experimentally in the present auditing study, and controlled manipulations such as region masking or intensity normalization would be required to move from association to mechanism.

Our mitigation strategy, utilizing group-balanced sampling and adversarial debiasing, preserved global performance while attenuating the specificity/FPR disparity, reducing the excess false-positive burden for female patients. Ablation analysis (Table S2) confirms that adversarial debiasing was the primary driver for gap reduction, independently achieving near-parity (+ 0.003 AUROC gap), whereas balanced sampling alone only partially mitigated the disparity (+ 0.027 gap). These findings suggest that baseline disparities may have been partially exacerbated by the underlying training set imbalance (57% male vs. 43% female; see Table S1), while the Grad-CAM findings raise the possibility of additional subgroup-correlated non-pulmonary image features contributing to model behavior. By enforcing equal exposure to subgroups via balanced sampling, our mitigation counteracted the representation bias that likely contributed to the poorer specificity in the underrepresented female cohort. At the same time, sensitivity gains were larger in males. This illustrates a key point for clinical fairness: different fairness criteria can move in opposite directions under a fixed operating point. Therefore, mitigation should be evaluated across multiple error types and calibration, rather than relying on a single metric. Crucially, the post-mitigation widening of the sensitivity gap illustrates the inherent theoretical trade-off in fairness optimization. The post-mitigation model should therefore not be interpreted as categorically fairer across all clinical objectives. Rather, mitigation shifted the error profile: it reduced the disproportionate false-positive burden among female patients, while increasing the sensitivity-related gap. The clinical acceptability of this trade-off depends on the intended use case. In triage- or screening-oriented applications, missed pneumonia cases may be more consequential and sensitivity may be prioritized. In contrast, in decision-support settings where false-positive alerts can trigger additional imaging, antibiotic exposure, or avoidable admissions, reducing false-positive disparities may be clinically valuable. These findings underscore that fairness mitigation should be evaluated against a prespecified clinical operating objective rather than a single fairness metric.

Several limitations are important for interpretation and directly address potential confounding. First, this study was based on a single public challenge dataset. Although the fixed sex- and label-balanced test cohort enabled controlled subgroup comparisons, it cannot establish whether the observed sex-specific differences generalize beyond the RSNA Pneumonia Detection Challenge dataset. The dataset is a curated subset of the NIH ChestX-ray8 repository^25^, and labels and bounding boxes reflect the challenge-specific annotation process. Dataset-specific acquisition factors, site- or device-related signatures, preprocessing pipelines, projection differences, image quality, and disease prevalence may be correlated with recorded sex and could influence both model performance and Grad-CAM localization patterns^21^. Consequently, the observed sex differences should be interpreted as dataset-specific signals requiring external validation in independent, preferably multicenter CXR cohorts with harmonized clinical labels and acquisition metadata. Second, sex was derived from binary DICOM metadata and does not capture gender identity; reporting frameworks encourage clearer separation and reporting of sex and gender variables where possible^26^. Third, segmentation-based explainability metrics depend on mask quality. Anatomical and surgical factors that alter soft-tissue attenuation, such as breast implants, mastectomy, or breast reduction, were not available in the dataset and could influence both performance and attention patterns. Furthermore, the balanced 50% pneumonia prevalence in the fixed test cohort differs from real-world clinical prevalence. At lower prevalence, the absolute false-positive burden and any sex-specific disparity in false-positive rates would be expected to increase, and the magnitude of reported rate-based gaps should therefore be interpreted in the context of the evaluation prevalence rather than as direct estimates of clinical impact.

In summary, this work provides insights into sex-stratified fairness auditing that connects subgroup error profiles with quantitative, anatomically grounded explainability. While absolute Grad-CAM localization should be interpreted cautiously given known limitations, the observed sex differences in attention distribution, together with error disparities, support the use of quantitative XAI as a complementary tool for identifying subgroup-dependent attention patterns that may indicate shortcut-prone model behavior and guiding mitigation in chest radiograph AI.

## Methods

### Dataset and preprocessing

We used the RSNA Pneumonia Detection Challenge dataset, comprising 26,684 frontal adult chest radiographs with expert-verified pneumonia labels and expert-annotated bounding boxes for positive cases. The RSNA dataset is a curated subset of the NIH ChestX-ray8 repository^25^. Recorded patient sex was extracted from DICOM metadata (PatientSex) and merged with image-level annotations to enable sex-stratified analyses.

All images were resized to 299 × 299 pixels, the native input resolution of InceptionV3, and normalized to the [− 1, 1] intensity range. To match the input specification of the InceptionV3 architecture, grayscale radiographs were replicated across three channels. Data augmentation was applied exclusively to the training set and consisted of random rotations, horizontal flips, and mild brightness and contrast adjustments.

### Train–validation–test split and fixed sex-balanced evaluation cohort

All splits were performed at the patient level to prevent information leakage. Prior to model development, we defined a fixed held-out test cohort (n = 1,000) balanced by recorded sex and pneumonia status (250 pneumonia-positive and 250 pneumonia-negative radiographs per sex; 50% pneumonia prevalence). This balanced cohort was used as the primary endpoint to ensure fair, like-for-like sex-stratified comparisons under an identical case mix. It was held constant across experiments and was not used for model selection, hyperparameter tuning, or threshold selection.

PR-AUC was computed on this label-balanced test cohort and therefore reflect performance under the 50% prevalence evaluation setting, alongside AUROC and operating-point sensitivity/specificity.

### Model architecture and training

An ImageNet-pretrained InceptionV3 convolutional neural network served as the feature extraction backbone^27^. A custom classification head consisting of global average pooling, batch normalization, dropout, fully connected layers with ReLU activations, and a sigmoid output layer was appended to produce pneumonia probability estimates. Models were optimized using the Adam optimizer with binary cross-entropy loss^28^.

Training followed a two-stage transfer learning protocol. First, the convolutional backbone was frozen and only the classification head was trained. Second, upper layers of the backbone were unfrozen and fine-tuned using a reduced learning rate. To assess robustness to random initialization and training stochasticity, the complete training pipeline was repeated across five prespecified random seeds. Final predictions were obtained using seed-ensembles formed by averaging predicted probabilities across seeds at the patient level. Across the five seeds, baseline discrimination was stable (AUROC 0.854 ± 0.003, balanced accuracy 0.775 ± 0.003, Brier 0.159 ± 0.002), and the seed-ensemble was used as the primary estimate to reduce initialization variance. The mitigation models followed the identical schedule and seeds.

### Operating point selection

For each training regime (baseline and fairness-mitigated), a single decision threshold was selected on the validation set using Youden’s J statistic, which maximizes the sum of sensitivity and specificity^29,30^. This single operating point was held fixed across all evaluations and both sexes, so that the rate-based error differences (TPR, FPR, and FNR gaps) reflect genuine subgroup differences rather than differences in threshold choice. Because error-rate metrics depend on the operating point, TPR/FPR/FNR gaps are reported at regime-specific validation-derived thresholds.

### Performance metrics, calibration, and sex gaps

Discrimination performance was quantified using the area under the receiver operating characteristic curve (AUROC) and the area under the precision–recall curve (PR-AUC). At the fixed operating point, we computed sensitivity, specificity, false-positive rate (FPR = 1 − specificity), false-negative rate (FNR = 1 − sensitivity), and balanced accuracy. Calibration was assessed using the Brier score, and further characterized using reliability diagrams and the expected calibration error (ECE; 10 quantile-based bins). Calibration intercept and slope were additionally reported to quantify systematic over-/underconfidence and spread. Calibration analyses were performed both overall and stratified by sex. To assess whether probability estimates could be improved without changing discrimination, post-hoc temperature scaling was fitted on the overall validation set and calibration was evaluated before and after scaling on the fixed test cohort; temperature scaling was used for calibration analyses only and does not affect rank-based discrimination metrics (e.g., AUROC). Because scaling was fitted globally, calibration effects can differ across subgroups; we therefore report sex-stratified calibration before and after scaling.

All metrics were reported both globally and stratified by sex. Sex gaps were defined as the difference between male and female performance (M − F). At the operating point, fairness gaps were reported as differences in TPR, FPR, and FNR between sexes.

### Explainability: Grad-CAM and quantitative in-lung attention

Model explainability was assessed using gradient-weighted class activation mapping (Grad-CAM)^22^. Grad-CAM heatmaps were computed from the final convolutional block of InceptionV3 (layer *mixed10*) by weighting feature maps with the global-average pooled gradients of the target logit, followed by ReLU rectification and min–max normalization to the [0, 1] range. Heatmaps were upsampled to the input image resolution using bilinear interpolation. To reduce variability arising from random initialization, Grad-CAM maps were aggregated at the ensemble level by averaging seed-specific heatmaps for each radiograph.

To quantify the anatomical plausibility of model attention, lung field segmentation masks were generated using pretrained anatomical segmentation models provided by the TorchXRayVision library^31^. Specifically, we used the ChestX-Det PSPNet anatomical segmentation model and extracted the “Left Lung” and “Right Lung” channels to form a binary lung mask^32^. In addition, we fused this mask with a TorchXRayVision U-Net lung segmentation model via logical union to improve robustness^31,33^. Masks were resampled to the model input resolution (299 × 299) using nearest-neighbor interpolation and post-processed using morphological closing/opening and retention of the two largest connected components to reduce boundary artifacts^31^. All test-set lung masks were visually inspected by M.H. (MD) for plausible lung coverage.

For quantitative analysis, Grad-CAM heatmaps were post-processed using a fixed configuration selected on a sex-balanced validation subset (n = 200 per sex) and then held constant for all test analyses. Post-processing consisted of Gaussian smoothing (5 × 5 kernel), percentile clipping at the 80th percentile, gamma correction (γ = 1.8), and morphological dilation (3-pixel kernel, one iteration). The in-lung activation fraction was defined as the proportion of total normalized Grad-CAM activation located within the lung segmentation mask. This metric was compared between sexes overall and stratified by prediction outcome (true positive, true negative, false positive, false negative) at the fixed operating point. Prediction outcomes were assigned by comparing each thresholded prediction with the reference pneumonia label. Among radiographs predicted as pneumonia, true positives carried a positive reference label and false positives a negative one. RSNA bounding boxes were used exclusively for illustrative visualization and not for quantitative localization evaluation.

### Fairness-aware mitigation

A combined fairness-aware mitigation strategy was evaluated while keeping the model backbone, preprocessing pipeline, training schedule, random seeds, and fixed test cohort identical to the baseline configuration. As part of this combined strategy, group-balanced sampling was implemented to equalize the representation of sex × label subgroups during training. We utilized a custom batch sampler that ensured every mini-batch explicitly contained equal proportions of the four subgroups (Male/Pneumonia-positive, Male/Pneumonia-negative, Female/Pneumonia-positive, Female/Pneumonia-negative), thereby preventing bias amplification driven by demographic or class imbalances.

In addition, adversarial debiasing was implemented by attaching an auxiliary sex-classification head to the shared feature representation (after layer mixed10) via a gradient-reversal layer (GRL). The GRL was placed on the global-average-pooled output of the final convolutional block (mixed10), so that the adversary acted on the same high-level features used by the pneumonia classifier and suppressed sex-predictive information at the representation level, while leaving lower convolutional layers unaffected. The adversary architecture consisted of a 128-unit dense layer with ReLU activation and dropout (p = 0.2), followed by a single sigmoid output neuron. The model was trained using a joint optimization objective defined as the sum of the primary task loss and the adversarial loss weighted by λadv (L = Ltask + λadv·Lsex). To stabilize training, we employed a dynamic weighting schedule for the gradient reversal strength (λadv): during the fine-tuning phase, λadv was linearly increased from 0.0 to 0.45 over the first 10 epochs (warm-up) and subsequently held constant until convergence (see Supplementary Listing S1 and Figure S7). To isolate the individual contributions of these techniques, we additionally conducted an ablation study evaluating group-balanced sampling and adversarial debiasing as independent components (see Table S2 in the Supplementary Material).

### Statistical analysis and uncertainty quantification

Primary estimates were reported for the seed-ensemble predictions obtained by averaging predicted probabilities across the five prespecified random seeds at the patient level. Uncertainty for all performance metrics, sex-stratified estimates, and gap measures was quantified using patient-level stratified bootstrap resampling with 10,000 iterations. Ninety-five percent confidence intervals were defined by the 2.5th and 97.5th percentiles of the bootstrap distributions. We adopted an estimation-based framework and did not prespecify a formal hypothesis test or fixed alpha. For difference and gap measures, including AUROC gaps, intervals excluding zero were interpreted as evidence of a difference, corresponding to a two-sided significance level of 5%. All analyses were implemented in Python using NumPy, pandas, and scikit-learn.

## Supplementary Information

Supplementary Information.

## Data Availability

All data analyzed in this study are publicly available via the RSNA Pneumonia Detection Challenge dataset. The dataset is derived from the NIH ChestX-ray dataset released by the NIH Clinical Center. NIH ChestX-ray dataset: https://nihcc.app.box.com/v/ChestXray-NIHCC. RSNA Pneumonia Detection Challenge dataset: https://www.rsna.org/education/ai-resources-and-training/ai-image-challenge/RSNA-Pneumonia-Detection-Challenge-2018. The code used for model training, evaluation, and explainability/fairness analyses is available from the corresponding author upon reasonable request.
