# Supplementary material for: Explainable AI for chest radiographs: sex-stratified fairness auditing in CNN-based pneumonia detection
Source: Sci Rep. 2026 Jul 20;16:22709. doi: 10.1038/s41598-026-62761-3 (PMC13385837; doi:10.1038/s41598-026-62761-3)
Supplement: Supplementary file 1 — Supplementary Information. [file 41598_2026_62761_MOESM1_ESM.pdf]

Supplements

Table S1. Demographics and distribution of the RSNA Pneumonia Detection Challenge dataset.

**Sex**	Count w/o pneumonia	Count w/ pneumonia	Total	Age (years) Mean ± SD
**Male**	11,656	3,510	15,166	46.4 ± 17.2
**Female**	9,016	2,502	11,518	47.7 ± 17.8
**Total**	20,672	6,012	26,684	47.0 ± 17.5

Table S2. Ablation Study of Fairness Mitigation Components Comparison of sex-stratified AUROC and performance gaps across individual mitigation components on the fixed test set (n = 1000; 250 pneumonia and 250 non-pneumonia cases per sex)**.** Results are reported as mean [95% CI] from 10,000 bootstrap iterations.

**Mitigation Component**	**Group**	**AUROC [95% CI]**	**Sex Gap (M−F) [95% CI]**
Balanced Sampling Only	Overall	0.849 [0.825 – 0.872]	+0.027 [-0.018 – 0.073]
	Male	0.864 [0.832 – 0.894]	
	Female	0.837 [0.803 – 0.870]	
Adversarial Only	Overall	0.846 [0.821 – 0.869]	+0.003 [-0.044 – 0.049]
	Male	0.849 [0.814 – 0.881]	
	Female	0.847 [0.812 – 0.879]	
Combined (Full Mitigation)	Overall	0.864 [0.843 – 0.885]	+0.026 [-0.018 – 0.069]
	Male	0.878 [0.847 – 0.906]	
	Female	0.851 [0.818 – 0.882]	

Supplementary Listing S1. Pseudocode of the gradient-reversal layer and dual-head adversarial debiasing (TensorFlow/Keras).

```
# Gradient-reversal layer (GRL) + dual-head adversarial debiasing
import tensorflow as tf
from tensorflow import keras
from tensorflow.keras import layers, models
from tensorflow.keras.applications import InceptionV3
@tf.custom_gradient
def _grad_reverse(x, lamb):
    def grad(dy):
        return -lamb * dy, tf.zeros_like(lamb)
    return x, grad
class GradientReversal(layers.Layer):
    def __init__(self, lamb=0.0, **kwargs):
        super().__init__(**kwargs)
        self.lamb = tf.Variable(float(lamb), trainable=False,
                                dtype=tf.float32, name="grl_lambda")
    def set_lambda(self, value):
        self.lamb.assign(float(value))
    def call(self, x):
        return _grad_reverse(x, self.lamb)
def build_encoder_and_heads(input_shape=(299, 299, 3)):
    base = InceptionV3(include_top=False, weights="imagenet",
                       input_shape=input_shape)
    feat = layers.GlobalAveragePooling2D()(base.output)   # after mixed10
    feat = layers.Dropout(0.3)(feat)                       # shared features
    # Task head (pneumonia)
    t = layers.Dense(256, activation="relu")(feat)
    t = layers.Dropout(0.2)(t)
    task_out = layers.Dense(1, activation="sigmoid", name="task_out")(t)
    # Adversarial sex head via GRL
    grl = GradientReversal(name="grl")
    z = grl(feat)
    z = layers.Dense(128, activation="relu")(z)
    z = layers.Dropout(0.2)(z)
    sex_out = layers.Dense(1, activation="sigmoid", name="sex_out")(z)
    for l in base.layers:
        l.trainable = False               # freeze backbone initially
```

```
    encoder   = models.Model(base.input, feat, name="encoder")
    task_head = models.Model(feat, task_out, name="task_head")
    sex_head  = models.Model(feat, sex_out, name="sex_head")
    return base, encoder, task_head, sex_head, grl
class AdvModel(keras.Model):
    """Joint loss: L = L_task + lambda_adv * L_sex (GRL on sex branch)."""
    def __init__(self, encoder, task_head, sex_head, grl, lambda_init=0.0):
        super().__init__()
        self.encoder, self.task_head = encoder, task_head
        self.sex_head, self.grl = sex_head, grl
        self.lambda_adv = tf.Variable(float(lambda_init), trainable=False,
                                      dtype=tf.float32)
        self.bce = keras.losses.BinaryCrossentropy()
    def set_lambda(self, value):
        self.lambda_adv.assign(float(value))
        self.grl.set_lambda(float(value))
    def train_step(self, data):
        x, y = data                        # y = [y_task, y_sex]
        y_task, y_sex = y[:, 0], y[:, 1]
        with tf.GradientTape() as tape:
            feat   = self.encoder(x, training=True)
            y_pred = tf.squeeze(self.task_head(feat, training=True), -1)
            s_pred = tf.squeeze(self.sex_head(feat, training=True), -1)
            l_task = tf.reduce_mean(self.bce(y_task, y_pred))
            l_sex  = tf.reduce_mean(self.bce(y_sex, s_pred))
            loss   = l_task + self.lambda_adv * l_sex
        grads = tape.gradient(loss, self.trainable_variables)
        self.optimizer.apply_gradients(zip(grads, self.trainable_variables))
        return {"loss": loss, "task_bce": l_task, "sex_bce": l_sex}
class LambdaWarmup(keras.callbacks.Callback):
    """Linear warm-up of lambda_adv from 0 to lambda_max over warmup_epochs."""
    def __init__(self, warmup_epochs, lambda_max):
        super().__init__()
        self.W, self.L = int(warmup_epochs), float(lambda_max)
    def on_epoch_begin(self, epoch, logs=None):
        lam = self.L * (epoch + 1) / self.W if epoch < self.W else self.L
        self.model.set_lambda(lam)
# Fine-tuning (Phase 2):
# base, enc, t_head, s_head, grl = build_encoder_and_heads()
# adv = AdvModel(enc, t_head, s_head, grl, lambda_init=0.0)
# adv.compile(optimizer=keras.optimizers.Adam(3e-5))
```

```
# adv.fit(ds_train, validation_data=ds_val, epochs=E,
#         callbacks=[LambdaWarmup(warmup_epochs=10, lambda_max=0.45)])
```

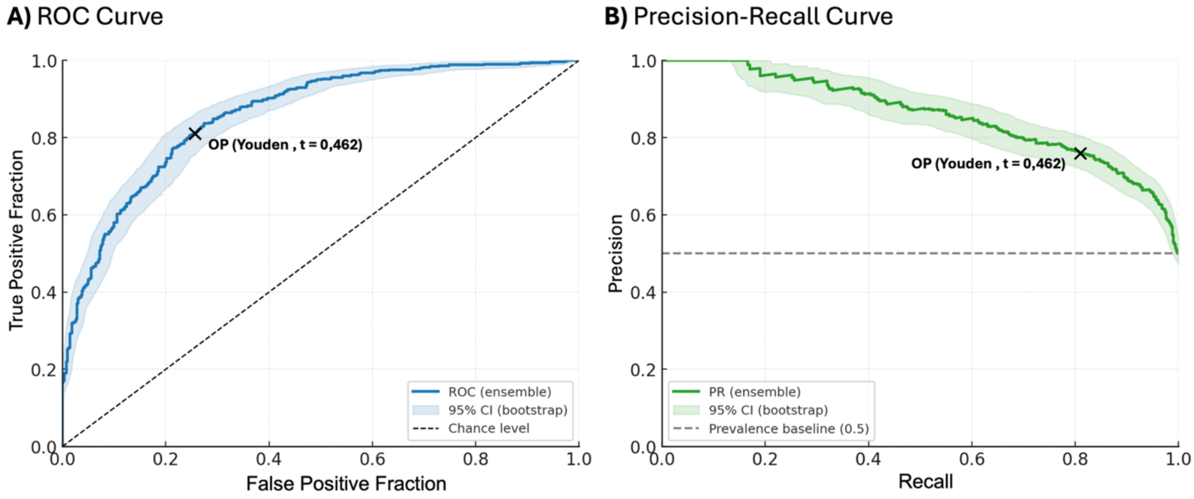

Figure S1. ROC and PR Curves of the Seed-Ensemble Model. Both panels show performance of the seed-ensemble model on the fixed balanced test set (n = 1000; 250 pneumonia and 250 non-pneumonia cases per sex), with shaded areas representing 95% confidence bands obtained via patient-level bootstrap resampling. The black marker denotes the fixed operating point chosen on the validation set using Youden's J statistic (t = 0.462). **(A)** ROC curve with the chance level shown as dashed diagonal. **(B)** PR curve with the prevalence baseline (0.5) shown as dashed horizontal line.

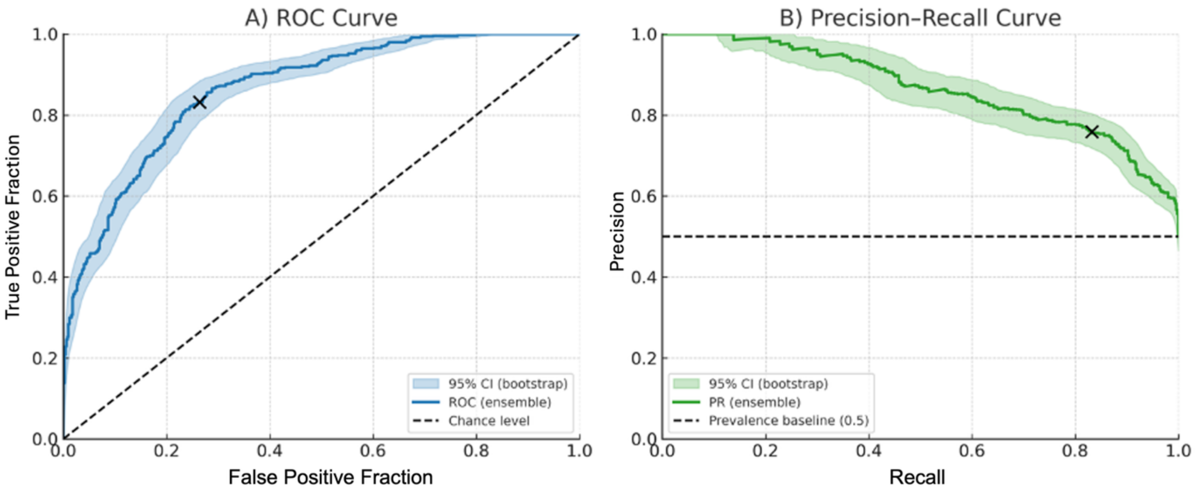

Figure S2: ROC and PR Curves of the Mitigation Ensemble Model. Both panels show performance of the ensemble model on the fixed balanced test set (n = 1,000; 250 pneumonia and 250 non-pneumonia cases per sex), with shaded areas representing 95% confidence bands obtained via patient-level bootstrap resampling. The black marker denotes the fixed operating point chosen on the validation set using Youden's J statistic (*t* = 0.425). **(A)** ROC curve with the chance level shown as dashed diagonal. **(B)** PR curve with the prevalence baseline (0.5) shown as dashed horizontal line.

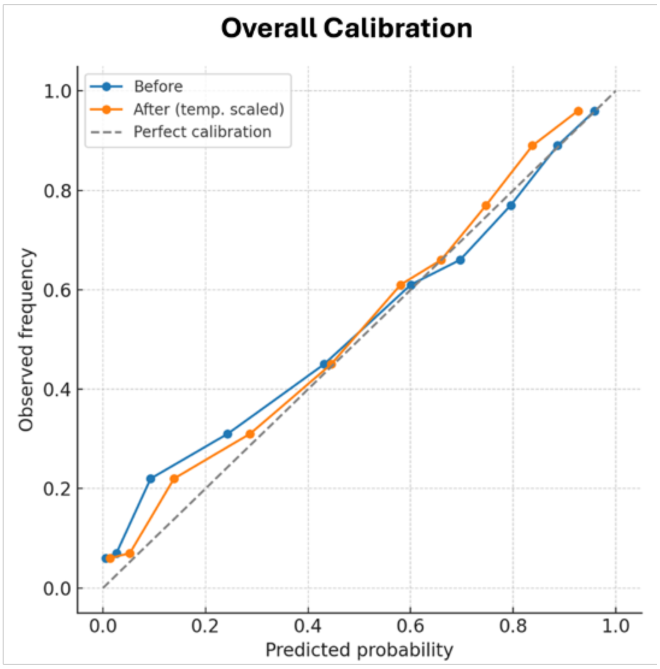

Figure S3: Calibration Plot of the Ensemble Model Before and After Temperature Scaling. Patients of the ensemble fixed balanced test set (n = 1000; 250 pneumonia and 250 non-pneumonia cases per sex) were grouped into 10 quantile-based bins according to predicted probability, and the mean predicted risk per bin was plotted against the observed pneumonia frequency. The dashed diagonal indicates perfect calibration. The blue curve shows the uncalibrated ensemble, which exhibited mild miscalibration (Brier score = 0.155 [0.142–0.169], ECE = 0.039 [0.032–0.071], slope = 0.73 [0.64–0.84]). The orange curve represents the model after temperature scaling, which improved calibration (Brier score = 0.154 [0.141–0.166], ECE = 0.033 [0.029–0.063], slope = 1.00 [0.87–1.15]), while the calibration intercept remained essentially unchanged (0.21 [0.05–0.38]).

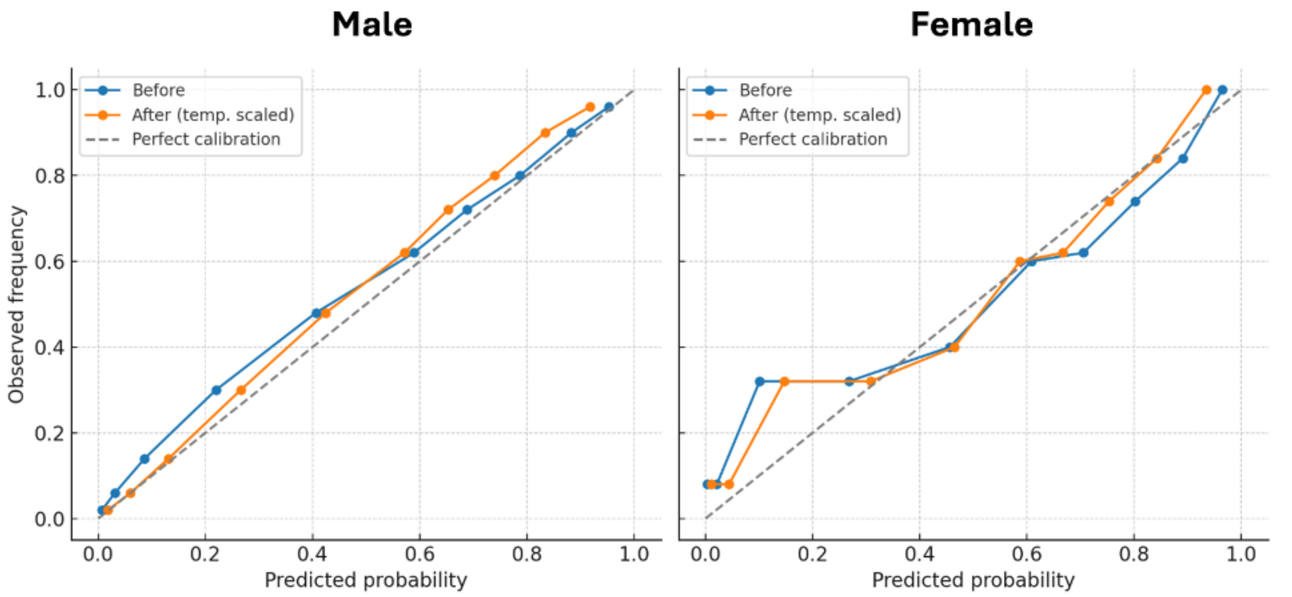

Figure S4: Sex-Stratified Calibration Plots of the Ensemble Model Before and After Temperature Scaling. The dashed diagonal indicates perfect calibration. Male cases were already close to the diagonal, with only marginal changes after calibration (Brier = 0.140 [0.123–0.158] → 0.141 [0.125–0.156]; ECE = 0.035 [0.028–0.080] → 0.045 [0.035–0.084]; slope = 0.87 [0.75–1.06] → 1.20 [1.02–1.43]). Female cases showed more pronounced miscalibration at baseline (Brier = 0.170 [0.149–0.193], ECE = 0.070 [0.051–0.112], slope = 0.61 [0.51–0.75]) and benefited from temperature scaling (Brier = 0.166 [0.149–0.185], ECE = 0.047 [0.041–0.094], slope = 0.84 [0.70–1.02]), reducing—but not fully eliminating—sex-specific differences in calibration.

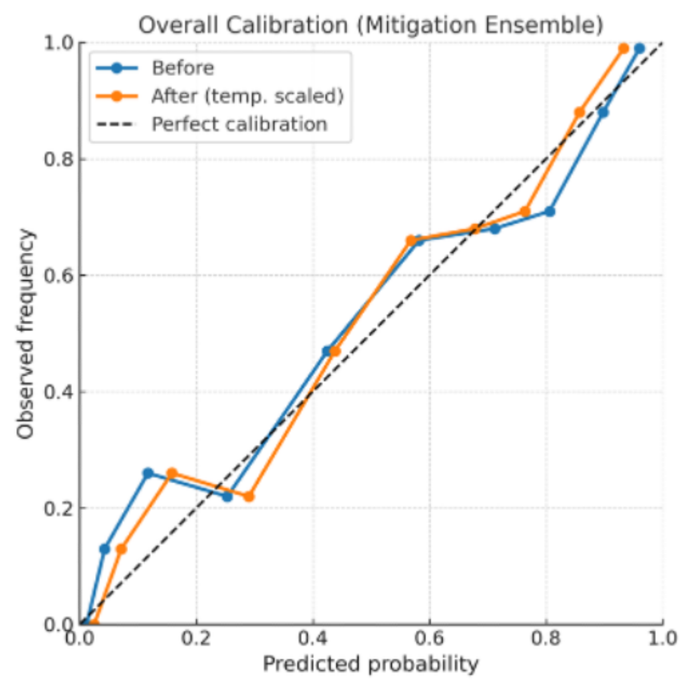

Figure S5: **Overall Calibration Plots of the Mitigation Ensemble Before and After Temperature Scaling.** The dashed diagonal indicates perfect calibration. At baseline, the mitigation ensemble showed mild undercalibration (Brier = 0.152 [0.138–0.166], ECE = 0.038 [0.031–0.071], slope = 0.78 [0.69–0.89]). After temperature scaling, calibration improved (Brier = 0.151 [0.137–0.165], ECE = 0.031 [0.027–0.062], slope = 0.95 [0.84–1.12]), while the intercept remained essentially unchanged (0.17 [−0.02–0.35]).

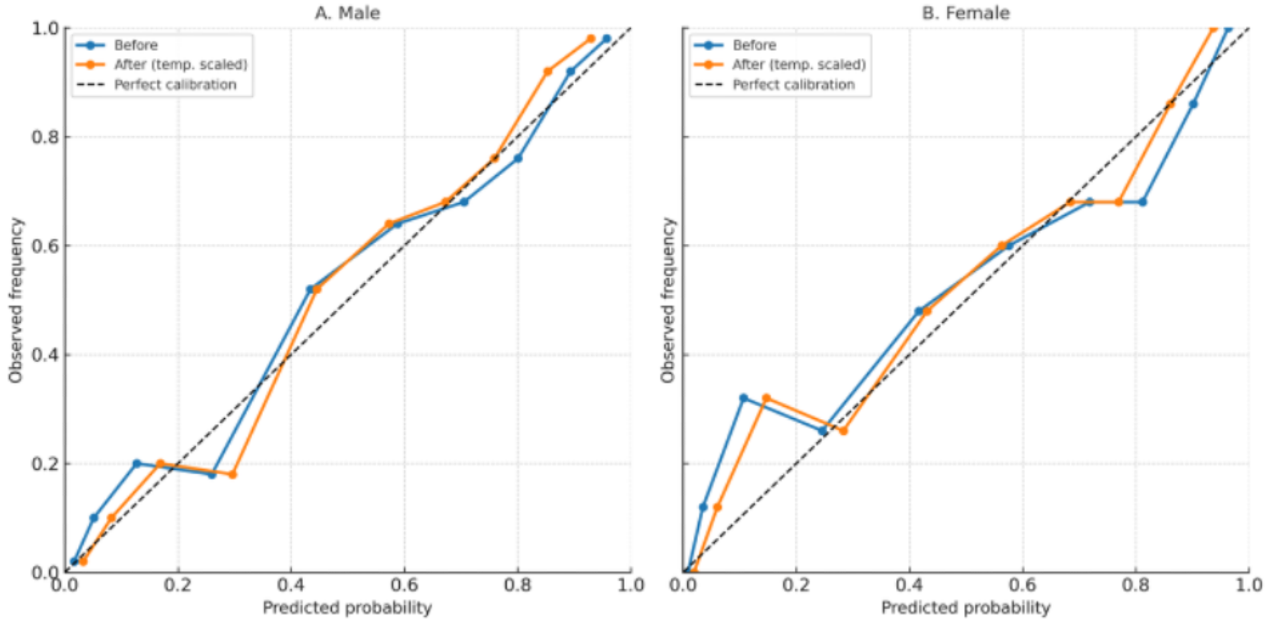

Figure S6: Sex-Stratified Calibration Plots of the Mitigation Ensemble Before and After Temperature Scaling. The dashed diagonal indicates perfect calibration. Male cases were already close to the diagonal, with only marginal changes after calibration (Brier = 0.142 [0.122–0.160] → 0.142 [0.125–0.159]; ECE = 0.046 [0.033–0.088] → 0.044 [0.036–0.088]; slope = 0.93 [0.80–1.12] → 1.06 [0.91–1.27]). Female cases showed more pronounced miscalibration at baseline (Brier = 0.163 [0.142–0.185], ECE = 0.066 [0.049–0.107], slope = 0.73 [0.63–0.88]) and benefited from temperature scaling (Brier = 0.161 [0.142–0.180], ECE = 0.055 [0.042–0.102], slope = 0.83 [0.72–0.98]), reducing—but not fully eliminating—sex-specific differences in calibration.

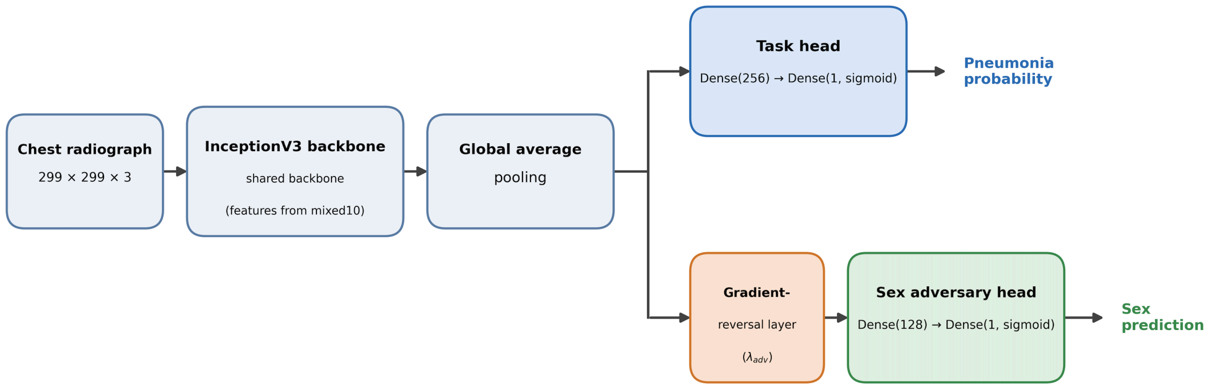

Figure S7. Schematic of the dual-head adversarial debiasing architecture. The shared ImageNet-pretrained InceptionV3 backbone is followed by global average pooling, and the resulting shared representation is split into two heads: a task head for pneumonia classification and an auxiliary sex-classification head connected through a gradient-reversal layer (GRL). During training the GRL drives the backbone to suppress sex-predictive information while the task head preserves pneumonia classification. Full implementation details are given in Supplementary Listing S1.
